# Multiphase preclinical assessment of a novel device to locate unintentionally retained surgical sharps: a proof-of-concept study

**DOI:** 10.1186/s13037-023-00359-8

**Published:** 2023-04-26

**Authors:** Hae Sung Kang, Jad Khoraki, Jessie Gie, Dielle Duval, Susan Haynes, Michael Siev, Jay Shah, Fernando Kim, Martin Mangino, Levi Procter, Riccardo Autorino, Samuel Weprin

**Affiliations:** 1 Department of Surgery, Virginia Commonwealth University Health, Richmond, VA USA; 2 Department of Urology, Stanford Health, Palo Alto, CA USA; 3 Department of Urology, Graves Gilbert Clinic, Bowling Green, KY USA; 4 Department of Urology, Yale School of Medicine, New Haven, CT USA; 5 Department of Urology, University of Colorado, Denver, CO USA; 6 New Jersey Urology, Summit Health, Cherry Hill, NJ USA

**Keywords:** Patient safety, Retained surgical sharps, Melzi Sharps Finder, Never event

## Abstract

**Background:**

Retained surgical sharps (RSS) is a “never event” that is preventable but may still occur despite of correct count and negative X-ray. This study assesses the feasibility of a novel device (“Melzi Sharps Finder®” or MSF) in effective detection of RSS.

**Methods:**

The first study consisted of determination of the presence of RSS or identification of RSS in an ex-vivo model (a container with hay in a laparoscopic trainer box). The second study consisted of determining presence of RSS in an in-vivo model (laparoscopy in live adult Yorkshire pigs) with 3 groups: C-arm, C-arm with MSF and MSF. The third study used similar apparatus though with laparotomy and included 2 groups: manual search and MSF.

**Results:**

In the first study, the MSF group had a higher rate of identification of a needle and decreased time to locate a needle versus control (98.1% vs. 22.0%, p < 0.001; 1.64 min ± 1.12vs. 3.34 min ± 1.28, p < 0.001). It also had increased accuracy of determining the presence of a needle and decreased time to reach this decision (100% vs. 58.8%, p < 0.001; 1.69 min ± 1.43 vs. 4.89 min ± 0.63, p < 0.001). In-the second study, the accuracy of determining the presence of a needle and time to reach this decision were comparable in each group (88.9% vs. 100% vs. 84.5%, p < 0.49; 2.2 min ± 2.2 vs. 2.7 min ± 2.1vs. 2.8 min ± 1.7, p = 0.68). In the third study, MSF group had higher accuracy in determining the presence of a needle and decreased time to reach this decision than the control (97.0% vs. 46.7%, p < 0.001; 2.0 min ± 1.5 vs. 3.9 min ± 1.4; p < 0.001). Multivariable analysis showed that MSF use was independently associated with an accurate determination of the presence of a needle (OR 12.1, p < 0.001).

**Conclusions:**

The use of MSF in this study’s RSS models facilitated the determination of presence and localization of RSS as shown by the increased rate of identification of a needle, decreased time to identification and higher accuracy in determining the presence of a needle. This device may be used in conjunction with radiography as it gives live visual and auditory feedback for users during the search for RSS.

## Introduction

A retained surgical item (RSI), which is defined as an unintended retention of a foreign object in the operative field, is a serious event with potentially life-threatening complications. Accordingly, it is categorized under “never event” by the National Quality Forum. Since 2012 when the Joint Commission started publishing data on sentinel events, RSI has been the most frequently reported sentinel event [1, 2]. It is believed to occur once every 1,000 to 18,000 surgeries [3–7]. As a result, this issue has been brought to the forefront of national health care policy. Accordingly, there has been a nation-wide effort to increase vigilance to prevent RSI with improved protocols, policy changes and new devices. However, despite of these efforts, it remains the most common cause of sentinel events in 2019 while the incidence of retained surgical sharp (RSS) continues to increase [2].

Every RSI has a significant cost, both to the hospital system and the patient. If recognized during the surgery, it may significantly increase the anesthesia time while increasing the risk of iatrogenic injury during the search. If recognized after, it requires an additional surgery to retrieve the item. In fact, one large case report suggests that RSIs are responsible for approximately 70% of surgical re-interventions [8]. Patients with known RSI are twice as likely to have postoperative complications [9]. In addition, one study showed that the management of RSI-related complications extends hospital stay by 8 days in 59% of the patients [10]. Unrecognized RSI may lead to an acute or an insidious clinical presentation, depending on the item and the patient factors. The clinical significance of retained needles is unclear, but there have been multiple case reports of complications such as organ injury such as bowel perforation, pneumothorax and chronic pain [11–13].

A RSS is defined as a lost sharp, most commonly a needle, not recovered prior to the patient leaving the operating room. Currently, most hospitals employ an RSS protocol that consists of strict needle counts after each case and utilization of radiography if the counts are inconsistent. However, correct counts do not necessarily mean an absence of RSS as one study found that 88% of RSI events occurred despite of a correct count [14, 15]. In addition, needles are the most commonly miscounted item [1, 9, 16]. Radiography also has its limits as well. Currently, plain film radiography is the imaging modality of choice to identify potential RSS. However, multiple studies suggest that plain film radiography may not be effective in identifying RSS if the needle is smaller than 17 mm; if the needle is smaller than 13 mm, it is unlikely to be found using radiography [17]. When evaluating the prolonged operative time associated with the use of X-ray, the added costs and radiation exposure to the patient, X-ray appears to be a poor choice for identifying RSS for smaller needles. In addition, plain film cannot provide live feedback during an ongoing search when the needle may shift positions during the search.

**Fig. 1 A/1B/1 C:**
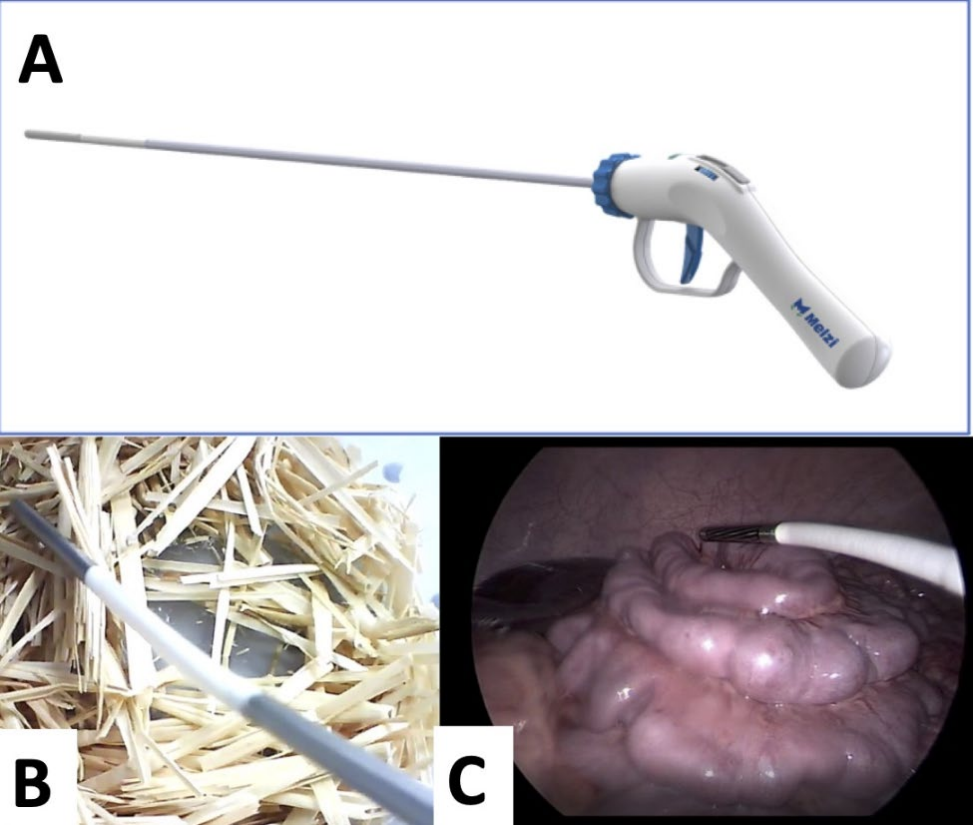
The device shown (1 A) can be used in both minimally invasive and open surgeries. Its flexible tip detects metallic objects and provides live visual and auditory feedback as a guide for the user. 1B and 1 C are pictures of MSF in use during the ex-vivo and in-vivo studies

In an attempt to increase the ability to find surgical sharps not detectable via X-ray, The Melzi Sharps Finder® (MSF) was developed (Fig. 1). MSF is a new technology that aims to identify small changes in magnetic fields that would indicate the presence or absence of a surgical sharp. The detector can be used in minimally invasive surgery or open procedure in a systematic search for a lost metallic sharp, with the proposed ability to improve detection rates and increase the confidence that there are no retained metallic sharps after a negative search. Here we present the first multiphase study evaluating the efficacy of this device in ex-vivo and in-vivo models of RSS.

## Methods

### MSF device

The MSF (Fig. 1a) is an FDA-approved single use device designed for use in minimally invasive surgery (MIS) and open surgery to detect miscounted and lost surgical needles as well as other metallic foreign bodies. The tip of MSF detects disturbances in magnetic fields, such as those caused by the presence of ferritic metals and translates these disturbances into auditory and visual signals to guide users to the location of the metallic body. The pitch of the sound and the frequency of the flashing light in the instrument give the users an idea of the proximity of the tip of the MSF to the metallic object. We performed *ex-vivo* and *in-vivo* studies to evaluate the utility of the MSF in (1) determining the presence of a needle in the abdomen after a miscount, and (2) locating a lost needle known to be in the abdomen.

### Ex-vivo evaluation (laparoscopic trainer box)

The MSF was evaluated in a “needle in a haystack” setting, in which suture needles were hidden in a laparoscopic trainer box filled with hay. Three users, consisting of one PGY-3 general surgery resident and two PGY-4 urology residents, were instructed on the basics of how to operate the device, but were not trained in how to perform a search. The users were allowed to develop their own method to search for the needle.

#### Clinical Scenario 1: Locating a known lost sharp

A 13 mm needle was hidden in a 10 g stack of hay in a 12.7 × 12.7 × 5.1 cm^3^ container and then placed into a Laparoscopic Trainer Box (Ethicon, Raritan, New Jersey USA). Users were randomly assigned to the control or device group in a 1:1 fashion via a random number generator for each study and were blinded to the needle location. The control group was allowed to use one grasper and a free hand to navigate through the hay in the container while the device group was given MSF in place of a grasper. The users were allowed to use their hands to move the hay or the container. The suture needle was considered found when directly visualized on the camera in the Laparoscopic Trainer Box (Fig. 1b) and the time was recorded. The primary endpoint was the user’s ability to locate the needle before the end of each study. The secondary endpoint was the time to find the needle. The user was given a maximum of 5 min to find the needle.

#### Clinical Scenario 2: Determining the presence of a sharp

Given the same apparatus, needle and MSF, the same users were asked to determine if a needle was present in the hay filled container. They were not allowed to move the hay or the container with their free hand. Each study was randomized to absence or presence of a needle via a random number generator. The primary endpoint was the user’s ability to successfully state at the end of each study whether a needle was present. The secondary endpoint was the time at which a user felt they could accurately determine the presence of needle. The user was given maximum of 5 min to decide.

### In-vivo evaluation (porcine animal model)

The MSF was evaluated in an in vivo porcine model (Fig. 1c). Live pigs were given intramuscular ketamine (20 mg/kg) with xylazine (2 mg/kg) and intravenous propofol (2-3 mg/kg) for anesthesia induction, and then intubated and ventilated with room air. Anesthesia was maintained with isoflurane (1–2%). The pig was positioned supine on a standard operating room table. After gaining access to the abdomen via either a laparoscopic or laparotomy approach, suture needles were hidden in different quadrants of the abdomen. The time to determine the presence of the needle and the time to reach this decision were recorded for each study. The users consisted of general surgery and urology residents of variable training from PGY3 to PGY5 from three different institutions. Prior to the beginning of the study, they were instructed on the basics of how to operate the device but were not trained in how to perform a search. The users were initially allowed to develop their own method to search for the needle. In scenarios in which X-ray was used, a radiologist was present to interpret the images. These studies were approved by the Virginia Commonwealth University (AM10019) and University of California Irvine’s Institutional Animal Care and Use Committee (AUP-20-112).

#### Clinical Scenario 3 (laparoscopy)

Male Yorkshire pigs (n = 2, 40 kg) were anesthetized to a plane of surgical anesthesia. Laparoscopic access to the abdomen was obtained and the abdomen was insufflated to 15mmHg. Three 5 mm trocars were placed with a midline periumbilical camera and two assistant ports on either side of the midline. Similar to *Clinical Scenario 2*, users were asked to determine if the needle was present in the abdomen. Users were randomly assigned to one of three groups to make these determinations: (1) C-arm with manual search using laparoscopic instruments, (2) C-arm with MSF, and (3) MSF alone. Each trial was further randomized to needle size (9 mm versus 26 mm), needle location (divided into right upper quadrant, left upper quadrant, right lower quadrant, left lower quadrant, periumbilical, pelvis) and the presence of a needle (with a 2:1 ratio of present versus absent). Block randomization was performed to ensure equal sample sizes. The length of each trial was capped at 5 min. Primary endpoints included user accuracy in determination of presence of needle and time to determine the presence of needle. Prior to each trial, the user was informed of a needle miscount and the size of the missing needle. In arms 1 and 2, a C-arm was used to obtain an X-ray of the abdomen at the beginning of each trial. The C-arm was not able to image the entire abdomen in one field of view and so each quadrant of the abdomen was imaged in a sequential fashion. The user and a radiologist interpreted the images in real time. If a needle was identified in one quadrant, the remaining quadrants were not imaged. If either the user or the radiologist decided that a needle was present on X-ray, the user was then instructed to begin searching for the needle using laparoscopic instruments alone (group 1) or laparoscopic instruments and the MSF (group 2). The user was given maximum of 5 min from the time of X-ray to locate the needle. If the user and radiologist both decided that there was no needle seen on X-ray, the trial was concluded. The users in group 3 were given the MSF without X-ray to determine the presence of the needle and locate it. Of note, plain radiography was unavailable and so we utilized a C-arm device to take still images as a substitute form of radiography.

#### Clinical Scenario 4 (laparotomy)

Male Yorkshire pigs (n = 2, 40 kg) were anesthetized to a plane of surgical anesthesia. The abdomen was accessed with a midline laparotomy incision extending from the xyphoid process to the pubic symphysis. Users were randomized to two groups to determine if the needle was present in the abdomen, and to locate the needle if present: (1) manual search alone and (2) manual search with assistance of MSF. The trials were randomized regarding the presence of needle, needle size, needle location in the same fashion as Clinical Scenario 3. The user was given a maximum of 5 min to decide. In both scenarios 3 and 4, if a user in a non-MSF arm incorrectly decided that there was no needle present in the abdomen, they were then provided with MSF to continue their search. The time to recover the needle with the MSF was recorded separately. Additionally, a systematic search was used in a subset of laparotomy trials to determine if this would independently increase the user’s accuracy or time.

### Statistical analysis

Data were analyzed using IBM SPSS Statistics for Windows, Version 27.0 (IBM Corp., Armonk, NY). Chi square test and unpaired t test were used to compare outcomes between two groups while ANOVA was used to compare outcomes amongst three groups. Multivariate linear regression analysis was performed to correlate independent and dependent variables in this study. Statistical significance was determined when the P-value was less than 0.05.

## Results

### Ex-vivo evaluation (laparoscopic trainer box)

#### Clinical Scenario 1 (Table 1)

A total of 101 trials were performed for Clinical Scenario 1. The control group (n = 50) successfully located the needle in 11 (22.0%) trials, compared to a success rate of 98.1% in the device group (n = 51) (p < 0.001). In addition, in the trials when the needle was found, the device group located the needle significantly faster than the control group (time to find the needle: 1.64 min ± 1.12 vs. 3.34 min ± 1.28; p < 0.001).

**Table 1:** Ex-Vivo Phase Results

		Control Group	Study Group	P value
**Study 1** **(n = 101)**	Rate of identification (%)	22.0% (11/50)	98.1% (50/51)	P < 0.001
	Time to identification (min)	3.34 ± 1.28	1.64 ± 1.12	P < 0.001
**Study 2** **(n = 56)**	Accuracy of report (%)	58.8% (20/34)	100% (34/34)	P < 0.001
	Time to report (min)	4.89 ± 0.63	1.69 ± 1.43	P < 0.001

The study group had significantly increased rate of successful identification and decreased time to identification. In addition, the study group had significantly increased accuracy in determining the presence of a needle in the field and needed less time to make this decision.

#### Clinical Scenario 2 (Table 1)

The control group (n = 34) correctly determined the needle’s presence 58.8% of the time while the study group’s accuracy (n = 34) was 100% (p < 0.001). The study group’s time to determine the presence of the needle was significantly shorter than that of the control group (1.69 min ± 1.43 vs. 4.89 min ± 1.43, p < 0.001).

### In-vivo evaluation (porcine model)

A total of 101 trials were performed (laparotomy: n = 49; laparoscopy: n = 52). The size of the needle was 9 mm in 49 trials (48.5%) and 26 mm in 52 (51.5%) trials. The MSF was used in 77 trials (76.2%) and X-ray was utilized in 19 trials (18.8%). A needle was present in the abdomen in 66 trials (65.3%) and accurate determination of the presence of a needle was achieved in 86 trials (85.1%).

#### Clinical Scenario 3 (Table 2)

A total of 52 trials were performed with 9 trials in arm 1 (X-ray with manual search), 10 trials in arm 2 (X-ray and MSF), and 33 trials in arm 3 (MSF alone). The distribution of needle size, needle location, and the presence of a needle between arms were comparable. The accuracy and speed of determining the presence of the needle in each group was comparable.

**Table 2:** In-Vivo Laparoscopic Phase Results

		X-ray + manual search (n = 9)	X-ray + MSF (n = 10)	MSF alone (n = 33)	P value
**Needle size**	9 mm	4 (44.4%)	5 (50%)	16 (48.4%)	0.97
	26 mm	5 (55.6%)	5 (50%)	17 (51.6%)	
**Needle present**		7 (77.8%)	7 (70%)	21 (63.6%)	0.71
**Needle absent**		2 (22.2%)	3 (30%)	12 (36.3%)	
**Success: all**		8/9 (88.9%)	10/10 (100%)	28/33 (84.5%)	0.42
**Success if needle present**		6/7 (85.7%)	7/7 (100%)	20/21 (95.2%)	0.49
**Success if needle absent**		2/2 (100%)	3/3 (100%)	8/12 (66.7%)	0.34
**Time to determine (minutes)**		2.2 ± 2.2	2.7 ± 2.1	2.8 ± 1.7	0.68

There was no statistically significant outcome in comparing the three groups using this laparoscopic porcine model. The MSF was non-inferior to the X-ray groups in determining the presence of a needle.

#### Clinical Scenario 4 (Table 3)

A total of 49 trials were performed with two groups using the laparotomy porcine model: manual search group (n = 15) and MSF group (n = 34). The distribution of needle size, needle location, and the presence of a needle between the groups were comparable. The success rate of the MSF group was significantly greater than that of the manual search group (97% vs. 46.7%, p < 0.001). The time to determine the presence of a needle was also significantly lower in the MSF group (2.0 min ± 1.5 vs. 3.9 min ± 1.5, p < 0.001).

**Table 3:** In-Vivo Laparotomy Phase Results

		Manual search (n = 15)	MSF (n = 34)	P value
**Needle size**	9 mm	8 (53.3%)	16 (47.0%)	0.69
	26 mm	7 (46.7%)	18 (52.9%)	
**Needle present**		9 (60%)	22 (64.7%)	0.75
**Needle absent**		6 (40%)	12 (35.3%)	
**Success: all**		7/15 (46.7%)	33/34 (97.0%)	< 0.001
**Success if needle present**		2/9 (22.2%)	21/22 (95.4%)	0.002
**Success if needle absent**		5/6 (83.3%)	12/12 (100%)	0.15
**Time to determine (minutes)**		3.9 ± 1.4	2.0 ± 1.5	< 0.001

The MSF group had statistically significant higher rate of successful determination of presence of a needle and decreased time to reach this determination. The distribution of needle size, needle location, and the presence of a needle between the groups were comparable.

### MSF Use

When multivariable logistic regression analysis was performed controlling for surgical approach (laparotomy vs. laparoscopy), needle size, needle presence, needle location and C-arm use, the use of the MSF was independently associated with an accurate determination of the presence of a needle (Table 4). The use of the MSF and the X-ray was associated with a 12.1 and 10.4-fold-increase in the odds of successful determination, respectively.

**Table 4:** Multivariable analysis

	Odds ratio	95% CI	P value
**MSF**	12.1	3.3–44.3	< 0.001
**X-ray**	10.4	1.1–98.9	0.041

When numerous factors (surgical approach, needle size, needle presence and needle location) were account for from Clinical Scenarios 3 and 4, MSF and X-ray were independently associated with an accurate determination of the presence of a needle.

## Discussion

This study assessed the feasibility of using MSF to determine the presence of a miscounted needle and identify misplaced needle in the surgical field. MSF significantly improved the time to determine the presence of a needle and the time to find a lost needle in the both the *ex-vivo* laparoscopic model and the *in-vivo* porcine model of laparoscopy and laparotomy.

In Clinical Scenario 1, the instrument allowed the users to quickly process through the hay and locate the needle without any visual cues based on auditory and visual signal. As each user sifted through the hay, the device was specific enough to guide the user to the needle within millimeters using the changes in the signals. As a result, the success rate of identification of the needle was 96% and the average time to localize was less than two minutes. The difficulty of directly visualizing the needle in the haystack is demonstrated by the 22.0% rate of needle identification without the device. This rate likely would not have improved significantly even if the users were allowed more than five minutes.

Clinical Scenario 2 tested the user’s ability to accurately determine whether a needle was present in the haystack. The MSF group had 100% accuracy while taking less than 5 min to decide. It is worth noting that in most trials, the user made this determination based solely on the device’s auditory feedback without visualizing the needle. This is akin to the detection of RFID embedded sponges with a wand external to the patient [18]. Without the device, the user was entirely dependent on visualization of the needle, which proved to be inefficient.

This laparoscopic model simulated the difficulties encountered in finding a needle in a real surgical field: poor visualization, narrow optics, a large heterogenous space, and the potential for the needle to shift positions. Recognizing the inherent limitations of this *ex* vivo model, the MSF was further evaluated in live porcine models.

The *in-vivo* trials were designed to simulate an intraoperative needle miscount in a human patient. Our porcine model aimed to simulate clinical scenarios of RSS with anatomic similarity, dynamic location of the needle in midst of the search, interference of standard operating table with its metallic components, respiratory and cardiovascular variations and limitations of surgical approach (laparotomy and laparoscopy).

The *in-vivo* results suggest that MSF is non-inferior to C-arm in its accuracy of determining the presence of a needle. Groups that used MSF had a very high accuracy in determining the presence of a needle (overall 92%). When compared to manual search, MSF demonstrated a significant improvement in user accuracy (97% vs. 46.7%). MSF had a positive predictive value (needle present) of 95.4% and negative predictive value (needle absent) of 100%. The time to determine the presence of a needle was also significantly shorter when using the MSF by an average of almost two minutes. In addition, multivariable analysis demonstrated that MSF use is independently correlated with a significant increase in the accuracy of identifying a lost needle in the abdomen, and MSF use had a stronger correlation to accurate determination of needle presence than X-ray.

The time to determine the presence of a needle was similar between C-arm and MSF. However, this does not factor in the additional time needed to obtain and interpret an X-ray, though in our trials both the C-arm and radiologist interpretation were immediately available. Our trials also imaged the abdomen by quadrants with the option to obtain additional images as needed to better examine certain quadrants, which may increase its sensitivity by focusing the radiologist’s attention. This arrangement is likely unrealistic in a clinical setting. The delay in obtaining and interpreting an X-ray has significant impact as it lengthens anesthesia time and increases perioperative risk for the patient while also taking up valuable OR time for the staff and the health system. The use of MSF may help to eliminate this time by effectively ruling out presence of needle without waiting for the X-ray or its interpretation.

Though there have been significant technological advancements to prevent soft retained items such as the radiofrequency identification of sponges, there has not been a widespread adoption of devices to prevent or aid in finding an RSS. One study explored the use of UV fluoroscopy to retrieve fluorescent coated needles and found that using the device improved surgeon’s time to retrieve the needles [19]. However, this would require hospital wide implementation of exclusive use of fluorescent coated needles. Another study found that using a magnetic retriever significantly reduced the search time for a lost needle [20]. This device inherently carries a risk of causing additional injury to organs during the search, which may be preventing its widespread adoption.

Currently, in the case of a miscount, the patient is kept under anesthesia while the surgical team performs a manual search and/or awaits X-ray images to be obtained and interpreted by a radiologist. In recent survey data, this is estimated to take 31–40 min of additional time per miscount event [21]. The combination of undue patient risk from the exposure to additional anesthesia and radiation coupled with the significant OR delays can create a frustrating environment for the surgical team. This study demonstrates the feasibility of a FDA-approved device which may be valuable in both the prevention and identification of RSS in the surgical field.

This study has several limitations and shortcomings. included a limited sample size. It is possible that the results may have been different with more trials. However, our outcomes were consistent throughout both *ex-vivo* and *in-vivo* experiments and the statistical analysis was robust to demonstrate that MSF is effective in determining a presence of a needle and finding it, if needed.

Due to the nature of the device indiscriminately finding metallic objects, there was an unusual situation that required the research coordinator to intervene during one of the laparoscopic trials. Persistent false positive signals were found while searching through the pig’s stomach and small bowel, assumed to be due to the pig’s ingestion of bars of the cage and metal filings. As a result, this required adjustment of sensitivity of the device, which allowed the user to facilitate effective search for the needle. During the laparotomy trials, this was not an issue.

Because MSF is a user-operated device, the results from this study may be affected by human error. As previously stated, the users were not instructed in how to conduct an intra-abdominal cavity search with MSF. As a result, each user had a different approach to their search, and this may have been a confounder in this study. While beyond the scope of this study, the authors encourage the use of a systematic search when using MSF to maximize accuracy and efficiency. We recommend the following methodology:

1. Sweep the right upper quadrant, making sure to include the large area obscured by the liver, especially the posterior side of the liver and the hepatoduodenal ligament.
2. Search above the body of the stomach and the gastrocolic ligament.
3. Retract the stomach caudad and search the left upper quadrant including perisplenic region.
4. Sweep the right and left paracolic gutters.
5. Perform a pass over the bowel prior to manipulation. When running each portion of the bowel, sweep ahead of the area you are about to manipulate, beginning with the jejunum at the ligament of Treitz and running the bowel to the terminal ileum.
6. Perform a sweep of the lower mid-abdomen and pelvis making sure to arrive on either side of bladder.

In addition, when the users were surveyed regarding their experience with the device after the trials, they collectively felt that the use of a sweeping motion or making concentric enlarging circles while working in different quadrants was the most effective way to rule out a sharp in a specific area.

## Conclusion

MSF is an FDA-approved device designed for use in both minimally invasive and open surgeries to detect metallic objects in the surgical field. It gives live auditory and visual signals to guide users to the location of the metallic body. In this multiphase preclinical study, the use of MSF in *ex-vivo* and *in-vivo* models of RSS appears to facilitate determination of presence and localization of surgical sharps. The study groups with MSF resulted in increased accuracy of determination of presence of a needle in the surgical field, increased rate of successful identification of a lost needle and decreased time to its identification. Future clinical trials are needed to validate and corroborate the findings from this experimental pilot study.

## Data Availability

The data that support the findings of this study are available from the corresponding author upon reasonable request.

## Abbreviations

MSF: Melzi Sharps Finder
OR: Operating room
RSI: Retained surgical item
RSS: Retained surgical sharps
